# Biofilm extracellular DNA enhances mixed species biofilms of *Staphylococcus epidermidis* and *Candida albicans*

**DOI:** 10.1186/1471-2180-13-257

**Published:** 2013-11-14

**Authors:** Mohan Pammi, Rong Liang, John Hicks, Toni-Ann Mistretta, James Versalovic

**Affiliations:** 1 Section of Neonatology, Department of Pediatrics, Texas Children’s Hospital & Baylor College of Medicine, 6621, Fannin, MC: WT 6-104, Houston, TX 77030, USA; 2 Department of Pathology, Texas Children’s Hospital & Baylor College of Medicine, Houston, TX 77030, USA

**Keywords:** Staphylococcus, Candida, Biofilms, Microarray, Extracellular DNA, Autolysis, Polymicrobial, Mixed species

## Abstract

**Background:**

Polymicrobial infections are responsible for significant mortality and morbidity in adults and children. *Staphylococcus epidermidis* and *Candida albicans* are the most frequent combination of organisms isolated from polymicrobial infections. Vascular indwelling catheters are sites for mixed species biofilm formation and pose a significant risk for polymicrobial infections. We hypothesized that enhancement of biofilms in a mixed species environment increases patient mortality and morbidity.

**Results:**

Mixed species biofilms of *S. epidermidis* and *C. albicans* were evaluated *in vitro* and in a subcutaneous catheter infection model *in vivo*. Mixed species biofilms were enhanced compared to single species biofilms of either *S. epidermidis* or *C. albicans.* A mixed species environment increased catheter infection and increased dissemination of *S. epidermidis* in mice. Microarrays were used to explore differential gene expression of *S. epidermidis* in the mixed species biofilms. In mixed species biofilms, compared to single species *S. epidermidis* biofilms, 2.7% of *S. epidermidis* genes were upregulated and 6% were down regulated. Staphylococcal autolysis repressors *lrgA* and *lrgB* were down regulated 36-fold and 27-fold respectively. The role of biofilm extracellular DNA was investigated by quantitation and by evaluating the effects of DNAse in a concentration and time dependent manner. *S. epidermidis* specific eDNA was increased in mixed species biofilms and further confirmed by degradation with DNAse.

**Conclusions:**

Mixed-species biofilms are enhanced and associated with increased *S. epidermidis*-specific eDNA *in vitro* and greater systemic dissemination of *S. epidermidis in vivo*. Down regulation of the *lrg* operon, a repressor of autolysis, associated with increased eDNA suggests a possible role for bacterial autolysis in mixed species biofilms. Enhancement and systemic dissemination of *S. epidermidis* may explain adverse outcomes after clinical polymicrobial infections of *S. epidermidis* and *C. albicans*.

## Background

Polymicrobial bloodstream infections are commonly due to coagulase-negative Staphylococci (CoNS, most commonly *S. epidermidis*) and Candida species [1-3]. Candida infections are important nosocomial infections in intensive care units and approximately 25% of patients with candidemia also have an associated bacteremia [4-6]. Polymicrobial infections are associated with significantly worse clinical outcomes than monomicrobial infections [2,7,8]. Mortality due to polymicrobial infections is twice that of monomicrobial infections in non HIV infected adult patients, children and neonates [9-11]. Pediatric polymicrobial infections also increase length of intensive care, therapy, hospital stay and healthcare costs [2].

Although high mortality has been observed in animal models of polymicrobial infections of Staphylococci and Candida, the mechanisms for increased mortality and morbidity have not been fully elucidated [12-15]. *In vitro* interactions of *Candida albicans* and *S. epidermidis* in mixed species biofilms and decreased antimicrobial susceptibility have been reported [16,17]. Interactions of *S. epidermidis* with Candida in mixed species infections may influence gene expression that may lead to enhanced virulence, biofilm formation, biofilm dispersal and tissue pathology have not been well studied.

A significant risk factor for human polymicrobial infections is the presence of indwelling vascular catheters that are sites for mixed species biofilm formation [2]. Biofilms are structured three dimensional microbial communities that are attached to a surface and encased in an extracellular matrix (ECM), which comprises extracellular DNA (eDNA), polysaccharides and proteins [18]. eDNA is formed by release of bacterial genomic DNA mostly by cell lysis or less commonly by active excretion into the biofilm matrix in some bacteria (e.g. Gammaproteobacteria) [18]. Extracellular DNA of the biofilms facilitates the initial stage of adhesion to biomaterials, forms the structural backbone and acts as glue that promotes biofilm aggregation [19-21]. Clinically significant mixed species biofilms of the pathogens *S. epidermidis* and Candida and the specific role of eDNA in mixed species biofilms have not been investigated.

In this study, we investigated mixed species biofilms of *S. epidermidis* and *C. albicans*, both *in vitro*, and in a clinically relevant mouse model of catheter biofilm infection, *in vivo.* We evaluated genome-wide *S. epidermidis* transcriptional responses in mixed species biofilms with *C. albicans*, to evaluate alteration in gene expression that causes increased virulence and pathogenicity of mixed species infections. We identified the significant role of eDNA in the enhancement of mixed species biofilms that may explain adverse outcomes due to clinical polymicrobial infections.

## Results

### Mixed species biofilms are larger than single species biofilms of *S. epidermidis* and *C. albicans*

**Figure 1:**
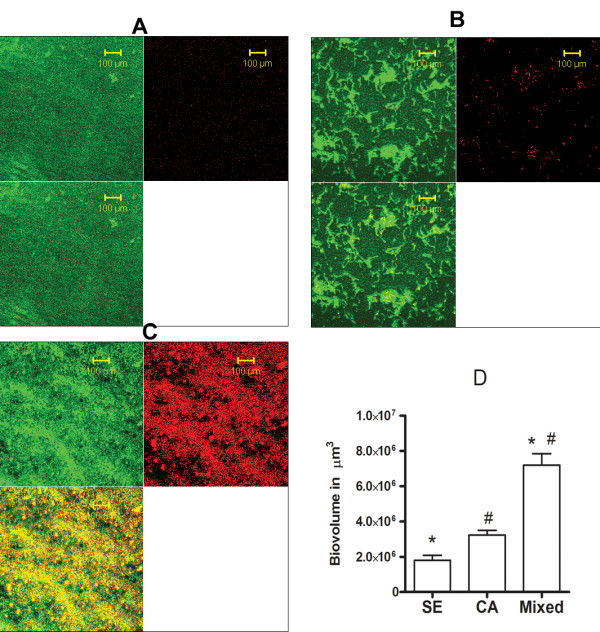
**Mixed species biofilms are larger than single species biofilms.** Twenty-four hour biofilms of *S. epidermidis* (SE) **(A)**, C. *albicans* (CA) **(B)** and mixed species biofilms (mixed) **(C)** were stained with LIVE/DEAD stain and examined by the Zeiss confocal microscope at 40× magnification. Scale bars measure 100 μm. For each biofilm, three channels are presented; green channel showing viable organisms, red channel showing non-viable organisms and the merged channel in that order respectively. Z-stacks of the biofilms at 1 μm intervals were analyzed by PHLIP software using MATLAB image processing toolbox and biovolume (μm^3^) compared **(D)**. Mixed species biofilms had significantly more biovolume than single species biofilms (*#p <0.05).

Representative confocal images of *S. epidermidis*, *C. albicans* and mixed species biofilms grown in microwell petridishes for 24 hr, stained with LIVE/DEAD, at 40× magnification, in the green, red and merged channels are presented in Figures 1A, 1B and 1C respectively. Mixed species biofilms that were developed using equal, half volumes of both organism suspensions (only half CFU/ml of each) grew more profusely than single species biofilms. Z-stacks of the biofilms at 1 μm intervals in the z axis at 40× magnification were analyzed by PHLIP software using MATLAB imaging toolbox. Biovolume of *S. epidermidis* (SE), *C. albicans* (CA) and mixed species biofilms (n = 6 each) are represented in Figure 1D. Biovolume of mixed species biofilms was significantly increased when compared to single species biofilms of either *S. epidermidis* or *C. albicans.*

### Scanning electron microscopy of explanted catheter segments confirms catheter biofilm infection *in vivo*

**Figure 2:**
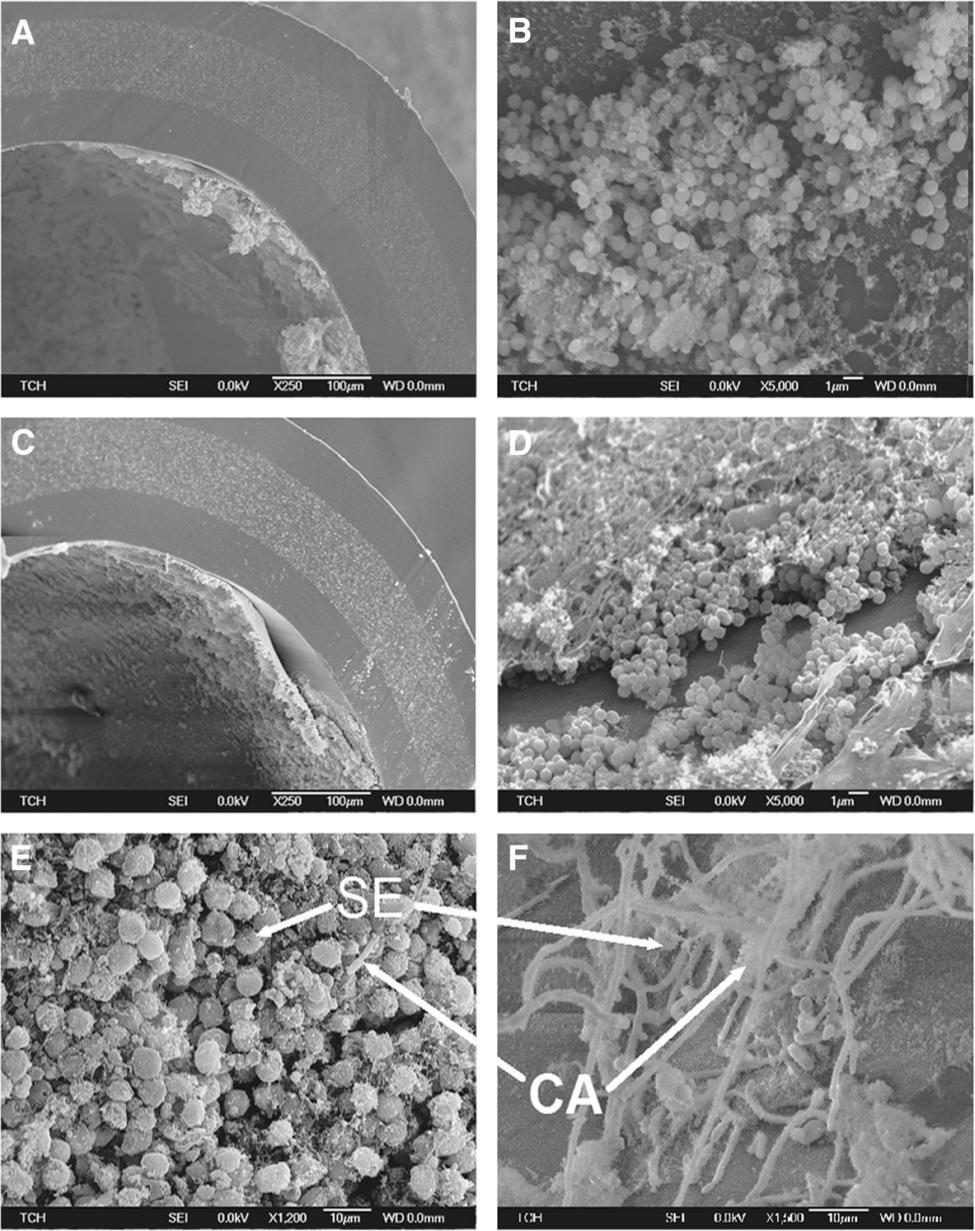
**Electron micrographs confirm catheter biofilms in the mouse model of subcutaneous catheter infection.** Subcutaneous catheter segments explanted on day 8 of infection were examined by scanning electron microscopy. Electron micrographs of *S. epidermidis* biofilm infection **(A and B)** and mixed-species biofilm infection **(C, D and E)** confirm biofilm formation on catheters *in vivo*. Mixed species biofilms where predominance of *S. epidermidis* (Figure 2**E**) and *C. albicans* (Figure 2**F**) are labeled for *S. epidermidis* (SE) and *C. albicans* hyphae (CA).

Scanning electron microscopy (SEM) of explanted catheter segments from mice on day 8 of insertion confirms catheter biofilm formation in the subcutaneous catheter model of biofilm infection. When examined using 250× magnification, *S. epidermidis* (Figure 2A, 2B) and mixed-species biofilms (Figure 2C, 2D) are seen coating the luminal surface of the catheter. *S. epidermidis* biofilms (Figure 2B) when examined at 5000× magnification, reveal grape-like clusters of Staphylococci. Mixed species biofilms have more organisms and extracellular material compared to single species *S. epidermidis* biofilms (Figure 2D). Candida hypha and *S. epidermidis* in mixed species biofilms are presented and labeled in Figure 2E and Figure 2F.

### Evidence for increased catheter infection and dissemination of *S. epidermidis* in mixed-species biofilm infection in a subcutaneous catheter model

**Figure 3:**
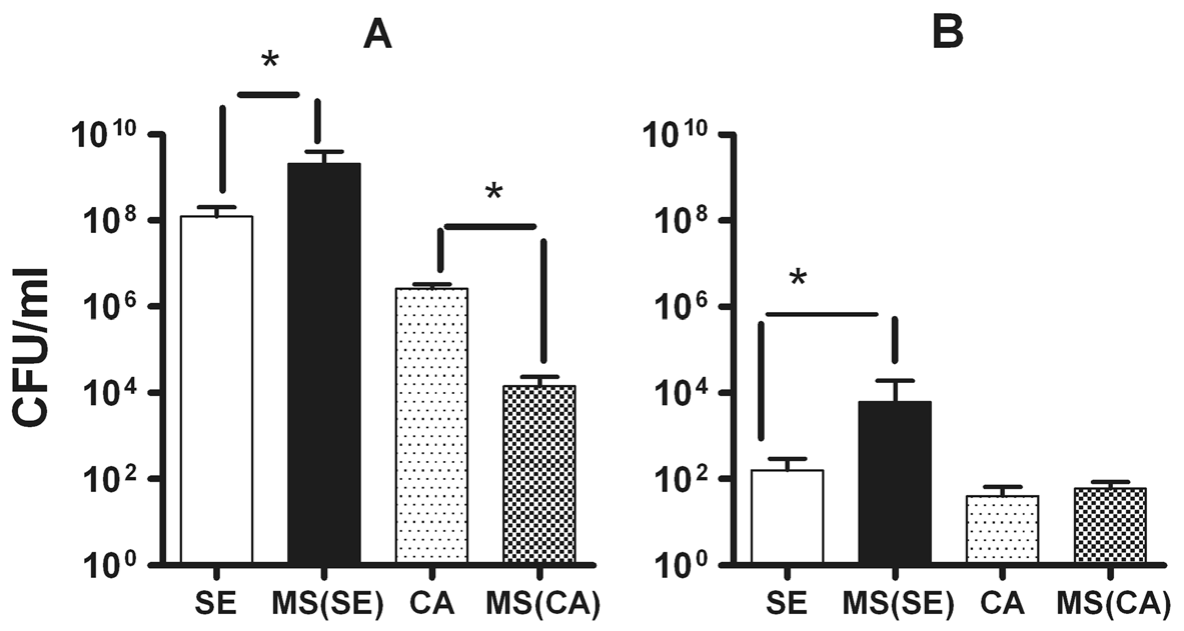
**Mixed species biofilms facilitate *S. epidermidis *infection and blood dissemination in the subcutaneous catheter biofilm model in mice.** Figure 3**A** depicts catheter CFU/ml and Figure 3**B** blood CFU/ml (systemic dissemination) of *S. epidermidis* and *C. albicans* in single species and in mixed species infections. *S. epidermidis* CFU/ml in mixed species infection was significantly greater than single species *S. epidermidis* infection both in catheters and in blood (p < 0.05). *C. albicans* CFU/ml from the catheter was significantly lower in mixed species biofilms then single species candida biofilms but were similar in the blood after single and mixed-species infections. *S. epidermidis* (SE) biofilms (single species) are shown in white bars, *S. epidermidis* in mixed species biofilms (Mixed (SE)) in gray bars, *C. albicans* (CA) (single species) in grainy bars and *C. albicans* in mixed species biofilms (Mixed (CA) in (chequered bars).

Figure 3A depicts catheter CFU/ml and Figure 3B blood CFU/ml (systemic dissemination) of *S. epidermidis* and *C. albicans* in single species and mixed species biofilm infections. Increased catheter biofilm formation was evidenced by significantly higher mean number of viable *S. epidermidis* in mixed species infection (2.04 × 10^9^ CFU/ml) compared to single species *S. epidermidis* biofilm infection (1.22 × 10^8^ CFU/ml) (p < 0.05). This is all the more significant since the pre-insertion catheter CFU/ml in the mixed species infection before subcutaneous insertion in mice were 1.5 to 2 × 10^4^ CFU/ml of *S. epidermidis* compared to catheters incubated in single species *S. epidermidis* infection (3.5 to 4.5 × 10^5^ CFU/ml). Since the pre-insertion CFU/ml were lower in the mixed species infection compared to single species *S. epidermidis* infection, adhesion phase of the biofilm formation is not altered by the presence of *C. albicans*. However, presence of *C. albicans* in the preincubation solution increases *S. epidermidis* biofilm formation on catheters *in vivo* possibly by increased biofilm aggregation resulting in increase in CFU/ml (Figure 3A) and extracellular matrix. Mixed species environment also increased dispersal of *S. epidermidis* as evidenced by increased blood dissemination of *S. epidermidis* in mixed species infection (mean blood CFU/ml was 6.08 × 10^3^ CFU/ml in mixed species infection compared 1.6 × 10^2^ CFU/ml in single species *S. epidermidis* infection, p < 0.05). *C. albicans* blood CFU/ml was similar in single and mixed species infection even though the catheter CFU/ml of Candida was significantly less in mixed-species biofilms compared to single species Candida biofilms (Figure 3A and 3B).

### Genome-wide transcriptional changes in *S. epidermidis* in mixed species biofilms compared to single species *S. epidermidis* biofilms

**Figure 4:**
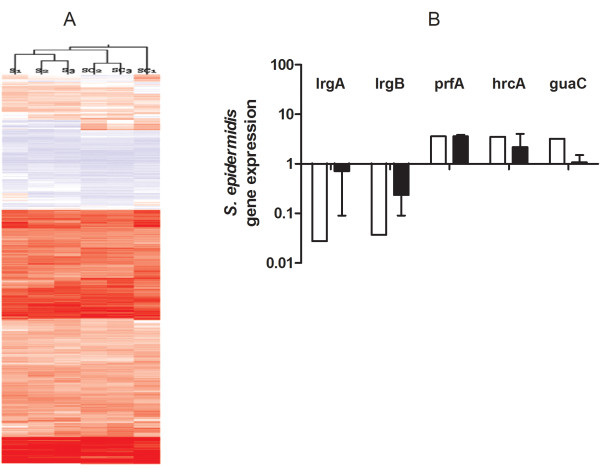
***S. epidermidis *transcriptome in mixed species biofilms and validation.** Figure 4**A** represents a heat map with hierarchal clustering of the samples. Red color indicates upregulation and light blue down regulation. S1, S2, S3 and SC1, SC2 and SC3 represent 3 biological replicates of single species *S. epidermidis* and mixed species biofilms respectively. Two down regulated genes (*lrgA and lrgB*) and 3 upregulated genes (*prfA, hrcA and guaC*) were evaluated for microarray validation (Figure 4**B**). Results for microarray are shown in white bars and real-time RT PCR in gray bars. Real-time RT PCR shows consistent results with microarray (p > 0.05 for each gene tested).

Microarray data have been deposited at the NCBI gene expression and hybridization data repository (http://www.ncbi.nlm.nih.gov/geo/), [GEO accession number GSE35438]. *S. epidermidis* gene expression in mixed species biofilms revealed 223 genes that changed ± 1.5 fold with an adjusted p value > 0.05. Upregulated *S. epidermidis* genes (2.7%) included s*arR* and the *hrcA* transcriptional regulators, heat shock protein *grpE*, genes involved in nucleic acid metabolism and other proteins (Additional file 1: Table S1). Down regulated *S. epidermidis* genes (6%) included the highly down-regulated *lrgA* and *lrgB* genes (repressors of autolysis, 36 fold and 27 fold change respectively), carbohydrate, amino acid and nucleotide metabolism, transporters and other proteins. Hierarchical clustering of data resulted in separation of samples of *S. epidermidis* and mixed-species biofilms*,* as expected (Figure 4A). The cluster analysis illustrates the quality of the biological replicates and the differential regulation between the two sample types. We selected three genes of known function that were upregulated > 3 fold (*prfA, hrcA* and *guaC*) and the two most down regulated genes (*lrgA* and *lrgB*) and performed real-time PCR to compare our microarray results (Figure 4B). Real-time PCR results were not statistically different from the microarray results for each of the genes evaluated (p > 0.05).

### Evidence for increased eDNA in mixed-species biofilms

**Figure 5:**
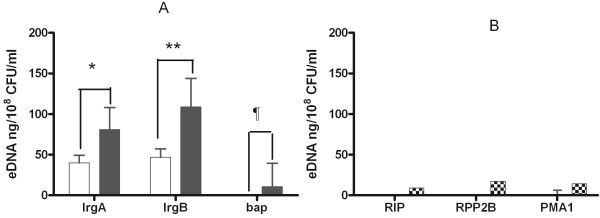
**Increased eDNA in the mixed-species biofilms confirmed by real-time RT PCR.** Biofilm matrix was extracted and eDNA was quantitated by real-time RT PCR using genomic DNA as standard. Primers for *S. epidermidis* genes (*lrg A, lrgB and bap*) were used to quantify the eDNA (Figure 5**A**). Staphylococcal eDNA was increased significantly in the mixed species biofilms compared to single species *S. epidermidis* biofilms (*, ** and ¶, p < 0.05). Candida gene specific primers (*RIP, RPP2B and PMA1*) were used to assess the contribution of eDNA by Candida in mixed species biofilms (Figure 5**B**). Candida specific eDNA was present in Candida biofilms, absent in *S. epidermidis* biofilms and negligible in mixed species biofilms. *S. epidermidis* biofilms are represented in white bars, mixed species biofilms in gray bars and Candida biofilms in chequered bars.

Quantification of the bacterial eDNA in the extracted biofilm matrix using *S. epidermidis* specific primers (*lrgA, lrgB and bap*) showed significantly increased bacterial eDNA in mixed-species biofilms of *S. epidermidis* and *C. albicans* compared to single species biofilms of *S. epidermidis* (Figure 5A). Extracted biofilm eDNA was normalized for CFU/ml of the initial organism suspension used to form the biofilms. In order to understand the contribution of eDNA from Candida, we assayed the eDNA with Candida chromosomal gene specific primers *RIP, RPP2B* and *PMA1* (Figure 5B). Candida specific eDNA was identified in single species Candida biofilms (< 30 ng/10^8^ CFU/ml), none in *S. epidermidis* single species biofilms and negligible in mixed species biofilms. This confirms the predominance of bacterial (Staphylococcal eDNA) in the extracellular matrix of mixed-species biofilms.

### Disrupting eDNA by DNAse decreases single and mixed-species biofilms

**Figure 6:**
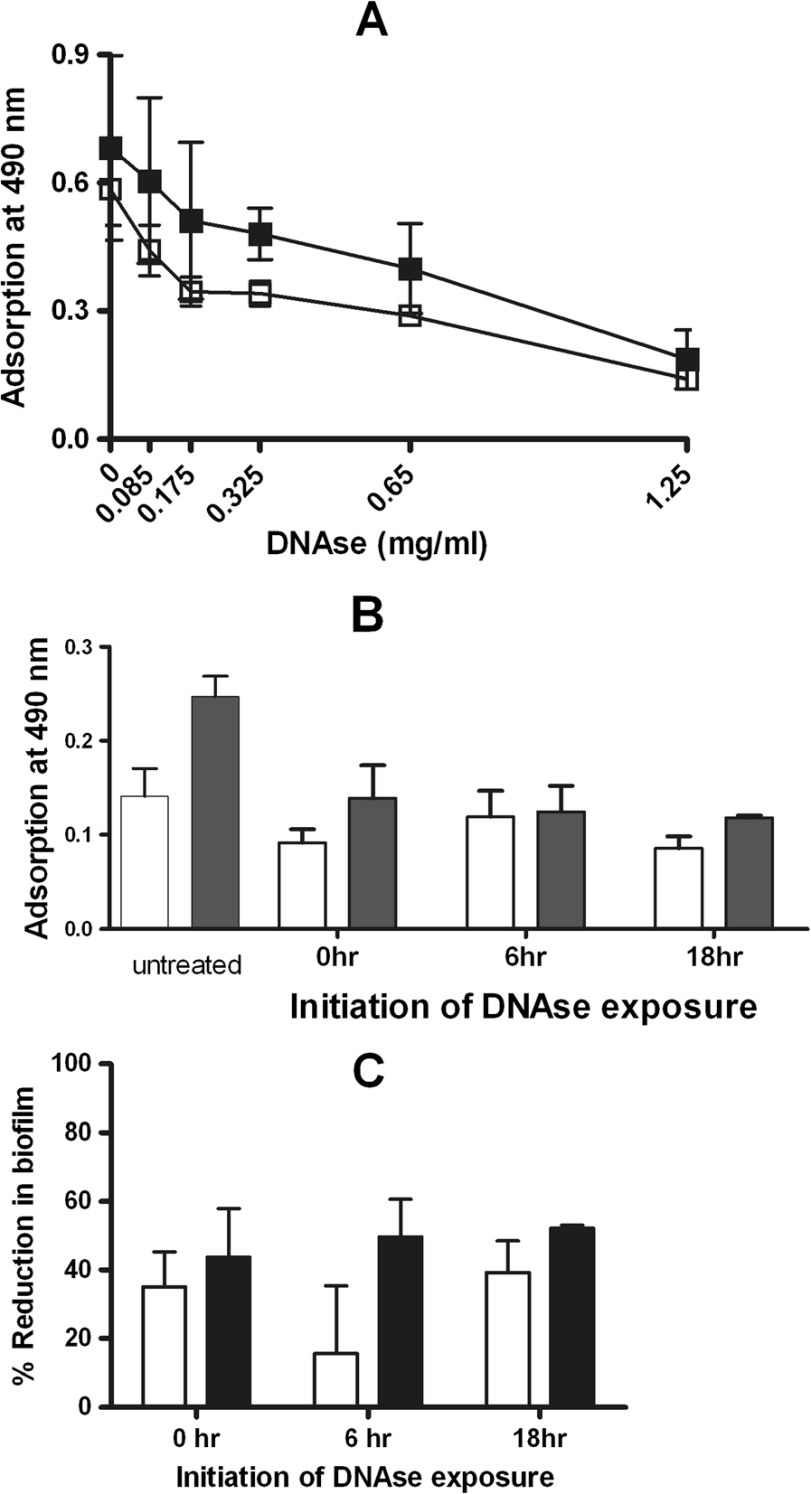
**Biofilm disruption of eDNA by DNAse.** Twenty four hr single species and mixed species biofilms were exposed to DNAse for 16 hr at concentrations from 0 (buffer) to 1.25 mg/ml (Figure 6**A**). Biofilm formation was significantly decreased by DNAse at 1.25 mg/ml compared to buffer (p < 0.05) and the biofilm disruption effect was concentration dependent. A time course experiment was performed by the addition of DNAse (0.65 mg/ml) at 0, 6 or 18 hrs and biofilm development continued till 24 hr and quantitated (Figure 6**B**). Both *S. epidermidis* and mixed species biofilm formation were significantly decreased (p < 0.05) after addition of DNAse at the three time-points of DNAse exposure. Percentage reduction in biofilms was more pronounced in mixed species biofilms compared to single species *S. epidermidis* biofilms (Figure 6**C**). *S. epidermidis* biofilms are represented in white squares and bars and mixed species biofilms in gray squares and bars.

We further confirmed the presence of eDNA by estimating the effects of DNA degradation on single and mixed species biofilms. DNAse I treatment for 16 hrs disrupted both single and mixed species biofilms of *S. epidermidis* significantly and in a concentration dependent manner compared to control biofilms (p < 0.05) (Figure 6A). In order to understand the effects of DNAse at different stages of biofilm formation, we exposed developing biofilms to DNAse (0.65 mg/ml) at 0, 6 and 18 hrs and developed the biofilms up to 24 hrs (Figure 6B). At all starting exposures, DNAse decreased biofilm formation at 24 hrs significantly compared to controls (p < 0.05). Percentage reduction in biofilms was more pronounced for mixed species biofilms compared to single species biofilms, indicating the higher eDNA content of the mixed species biofilms (Figure 6C).

## Discussion

We evaluated the morphology of mixed species biofilms of *S. epidermidis* and *C. albicans, in vitro*. We observed enhancement of biofilms in a mixed species environment. In a mouse subcutaneous catheter model of biofilm infection, we noted increased catheter infection and systemic dissemination of *S. epidermidis* in a mixed species environment. To further explore the reasons for increased pathogenicity of *S. epidermidis* in mixed species biofilm infections with *C. albicans*, we evaluated the transcriptome of *S. epidermidis* in a mixed species environment and found that the repressors of autolysis, *lrgA* and *lrgB* were highly down regulated. Down regulation of repressors of autolysis, is associated with increased eDNA in the biofilm matrix, possibly by increased bacterial autolysis. We confirmed the significance of increased biofilm eDNA by evaluating its degradation by DNAse.

Mixed species biofilms of *S. epidermidis* and *C. albicans* were significantly thicker and voluminous compared to single species biofilms of either organism *in vitro*. Increased thickness of mixed species biofilms can be due to increase in the number of organisms or increase in the extracellular matrix or possibly both. In mixed species biofilm infections *in vivo,* at 8 days of infection, we observed increase in catheter CFU/ml of *S. epidermidis* associated with blood dissemination. Mixed species biofilms *in vivo* may further be modified by environmental milieu e.g. conditioning of the catheter implants with host proteins that may increase biofilm adhesion and aggregation.

In mixed species biofilms of other bacteria with Candida species, bacterial association with hyphae predominates association with yeast cells [22,23]. Hogan *et al.* evaluated interactions of *Pseudomonas aeruginosa* and Candida, and found that *Pseudomonas aeruginosa* had a predilection for the hyphal form without affecting the yeast form of the fungus [22]. In studies of mixed species infections of *S. aureus* and *C. albicans*, similar to *P. aeruginosa*, adherence to the Candida hyphae was nearly 30-fold more than adherence to the yeast form of Candida [23]. In our experiments (data not shown) we found adherence of *S. epidermidis* to both yeasts and hyphae of Candida which may facilitate mixed species biofilms of these two organisms and partly contribute to the increased clinical frequency of mixed species biofilm infections of *C. albicans* and *S. epidermidis*. The yeast and hyphal forms of *C. albicans* may act as a scaffold on which biofilms of *S. epidermidis* are formed [23]. Candida infection is associated with tissue invasion by hyphae and it been hypothesized that staphylococcal tissue infection is facilitated by its association with Candida hyphae [23]. Synergistic effects of *C. albicans* and *S. epidermidis* have been reported by other investigators [16,17]. In mixed species biofilms of *C. albicans* and *S. epidermidis*, presence of slime producing strains of *S. epidermidis* decreases antifungal susceptibility related to decreased penetration of the fluconazole through the ECM and conversely the fungal cells protected slime negative *S. epidermidis* against vancomycin [16]. In an *in vitro* study of mixed species biofilms of *C. albicans* and *S. epidermidis*, enhanced the growth of *S. epidermidis* was observed [17].

We used a clinically relevant model of subcutaneous catheter biofilm infection to evaluate the clinical implications of mixed species biofilm infection [24]. In mixed species biofilms, catheter biofilm infection of *S. epidermidis* increased in the presence of *C. albicans*. Pre-insertion cultures revealed lower catheter infection of *S. epidermidis* in mixed species infection compared to single species *S. epidermidis* but on day 8 of insertion *in vivo*, we found increased catheter infection of *S. epidermidis* in the mixed species infection. This suggests that mixed species environment facilitates biofilm aggregation and not the initial phase of *S. epidermidis* adhesion to catheters. Enhanced biofilm aggregation was associated with enhanced dispersal that led to increased systemic dissemination of *S. epidermidis* in the mixed species infection. Increased virulence and mortality has been described in mouse models of dual infection with *C. albicans* and *S. aureus* but not with *S. epidermidis*[12-14]. Peters *et al.* using proteomic techniques found that the global transcriptional repressor of virulence was down regulated thereby increasing virulence of the dual species biofilms of *S. aureus* and *C. albicans*[23]. Enhancement of biofilms, increased catheter infection and dissemination of *S. epidermidis* in mixed species biofilms *in vivo* may partly explain clinical therapeutic failures and contribute to increased mortality and morbidity in polymicrobial infections.

We performed microarrays to delineate changes in staphylococcal gene expression that lead to increased catheter infection and dissemination in mixed species biofilms with *C. albicans*. We noted that the *lrg* operon comprising *lrgA* and *lrgB* was highly down regulated (36 fold and 27 fold change respectively) in mixed species biofilms. *Lrg* operon along with the *cidR* operon represents the molecular elements of programmed cell death or apoptosis in *Staphylococcus aureus*[25-27]. The *lrg* operon is a repressor of murein hydrolase activity that hydrolyzes components of the cell wall, involved in autolysis. Lrg protein has also been shown to affect antibiotic tolerance, biofilm formation (by release of eDNA which is a structural component of the biofilm) and acetoin production in *S. aureus*[25,26,28,29]. *Lrg* operon is regulated by the LytSR two component regulatory system in *S. aureus* and transcriptional regulators *agr* and *sar* that regulate virulence also influence the *lrg* operon [28,29]. Down regulation of the *lrg* operon (autolysis repressors) in mixed species biofilms is associated with enhanced release of eDNA possibly by autolysis [25,30]. Extracellular DNA plays a significant role in biofilm aggregation [18,19] and it is conceivable that increased eDNA enhances aggregation of mixed species biofilms of *S. epidermidis* and *C. albicans*. Most bacteria have cardiolipin synthases that convert bacterial membrane phosphatidyl glycerol to cardiolipin, during the transition from logarithmic phase to the stationary phase and may help survival during prolonged high salt stress conditions [31]. *S. aureus* and *S. epidermidis* have 2 ORFs *cls1* and *cls2*[32] and we found cardiolipin synthetase (*cls2*) was significantly down regulated. Other down regulated genes included those associated with carbohydrate, amino acid and nucleotide metabolism, transporters and other proteins. Biofilm as a whole may be metabolically less active compared to actively dividing planktonic organisms and that may explain the down regulation of metabolic processes and overall more down regulated genes (6%) than upregulated genes (2.7%) [33].

Genes upregulated in mixed species biofilms include transcriptional regulators (*sarR* and *hrcA* the heat inducible transcriptional repressor), genes associated with nucleic acid metabolism, some transporters and other proteins. *sarR* is known inhibitor of *sarA*, a transcriptional regulator that represses extracellular proteases and that may influence virulence determinants in *S. aureus*[34-36] but its role in *S. epidermidis* is not known. Therefore the net effect of *sarR* upregulation is to facilitate secretion of extracellular proteases that may function as virulence factors. Heat shock protein GrpE protein of the DnaK family of shock proteins is upregulated indicating an adaptive response to polymicrobial stress by *S. epidermidis* in mixed species biofilms. Adaptation to competition for iron in mixed species environments is facilitated by the increased transcription of transferrin receptor, which facilitates uptake of iron from human transferrin by a receptor-mediated energy dependent process [37,38]. Genes related to nucleic acid and glycerol metabolism (*guaC, purC, purM, glpD, apt and uraA*) were also upregulated.

We measured the eDNA content in the extracellular matrix of single and mixed-species biofilms and confirmed that *S. epidermidis* derived eDNA predominated in mixed species biofilms. Candida derived eDNA was barely detected indicating the predominant role for bacterial eDNA in the enhancement of mixed-species biofilms. Low Candida eDNA may be also partly due to decreased growth of Candida in mixed species biofilms. Indirectly, this indicates that bacterial autolysis, the most important mechanism for producing bacterial eDNA, is strongly implicated in the enhancement of mixed species biofilms.

We evaluated the effects of disrupting eDNA by DNAse on mature (24 hr) and developing single and mixed species biofilms of *S. epidermidis* and *C. albicans*. DNAse decreased biofilm metabolic activity (as measured by XTT method) by a concentration dependent manner in both single and mixed species biofilms. We also evaluated the effects of DNAse on a developing biofilms by initiating exposure to DNAse at different time points (0, 6 and 18 hrs). Exposure at earlier time-points would decrease adhesion of the microbial cells and exposure later would affect biofilm aggregation. We observed that DNAse decreased biofilm formation significantly at both adhesion and aggregation stages in biofilm development. The reduction in biofilm formation as a percentage of that of untreated biofilms was more pronounced in mixed species biofilms compared to single species biofilms, due to an increased eDNA content in the mixed species biofilms. Other investigators have found similar inhibiting effects of DNAse on biofilm adhesion and aggregation outlining the essential role of eDNA in biofilm development [39-41].

We confirmed increased eDNA in mixed species biofilms by quantitation of eDNA in the biofilm extracellular matrix. Increased eDNA in the biofilm matrix is probably caused by autolysis as active secretion of eDNA has not been reported in *S. epidermidis* biofilms. Staphylococcal biofilm aggregation is enhanced by eDNA and increased quantity of eDNA may explain the increased thickness of mixed-species biofilms. Significant down regulation of repressors of autolysis (*lrg* operon) also point to increased bacterial autolysis in mixed species biofilms. The *lrg* operon that represses murein hydrolase activity and thereby autolysis in *S. aureus* has not been studied in *S. epidermidis* so far. In *Staphylococcus aureus, cidA* and *lrgA* genes encode homologous hydrophobic proteins that function similar to bacteriophage coded holin (causes autolysis) and antiholin (inhibits autolysis), respectively. The *S. aureus cidB* and *lrgB* genes also encode homologous hydrophobic proteins, but their functions are unknown [42]. In a model proposed by Bayles et al., the LytSR two-component regulatory system senses decreases in cell membrane potential due to cell membrane damage and responds by inducing *lrgAB* transcription. The CidR protein, a LysR-type transcription regulator, enhances *cidABC* in response to carbohydrate metabolism that enhance murein hydrolase activity thereby enhancing autolysis [26,43]. *LrgAB* operon in *S. aureus* also influences penicillin (that causes cell lysis) tolerance [25]. In *S. epidermidis,* LytSR knockout strain exhibited decreased extracellular murein hydrolase activity and mildly increased biofilm formation but did not differ in Triton X-100 mediated autolysis or in murein hydrolase zymogram patterns from the parent strain [44]. Mutation of *SaeRS* (another two component signal system) in *S. epidermidis* increased autolysis and biofilm forming ability [45]. Association of autolysis and increased biofilm formation is also confirmed by studies on autolysin *atlE* in *S. epidermidis*[46]. Therefore, autolysis and release of eDNA has a significant role to play in Staphylococcal biofilm formation and enhancement of mixed species biofilms.

The limitations of the study include using a single clinical strain each of *S. epidermidis* and *C. albicans*. Findings of this study will have to be confirmed using multiple strains of *S. epidermidis* and *C. albicans*. The subcutaneous catheter biofilm infection in mice is an appropriate and reproducible model to evaluate foreign device biofilm infections *i.e.* pacemaker and shunt infections but an intravenous catheter model will be more appropriate for indwelling vascular catheter infections. Nevertheless the subcutaneous catheter model has been successfully used to study biofilm infections and to evaluate anti-biofilm strategies. In our microarray experiments, *S. epidermidis* probes on the microarray that might hybridize with Candida RNA were eliminated in the design of the microarray. Also, those probes that actually hybridized with Candida RNA were also eliminated from data analysis. It is possible that some transcriptome data was lost due to the elimination of Candida cross-reacting probes.

## Conclusions

Biofilms are enhanced in a mixed-species environment of *S. epidermidis* and *C. albicans* both *in vitro* and *in vivo.* Enhanced mixed-species biofilms are associated with increased *S. epidermidis*-specific eDNA *in vitro* and greater systemic dissemination of *S. epidermidis in vivo*. Down regulation of the *lrg* operon, a repressor of autolysis was associated with increased eDNA. We propose that bacterial autolysis may play a significant role in the enhancement of mixed species biofilms and which needs to be confirmed by mechanistic studies. Enhancement of biofilms and systemic dissemination of *S. epidermidis* in polymicrobial environments may explain increased clinical mortality and morbidity. Elucidation of polymicrobial interactions in mixed species biofilms may lead to novel strategies to treat human polymicrobial infections.

## Methods

### Organisms, strains and culture conditions

Human isolates of *S. epidermidis* (strain 1457) and *C. albicans* (strain ATCC 32354) were used in this study. *S. epidermidis* were incubated in tryptic soy broth (TSB) broth for 2 hr from overnight TSB agar plates. *C. albicans* was plated on Sabouraud’s dextrose agar (SDA) overnight and grown in Yeast Peptone Dextrose (YPD) broth for 4 hr. Both organisms were adjusted to an optical density (O.D.) of 0.3 in RPMI 1640 (10^7^ CFU/ml of *S. epidermidis* and 10^5^ CFU/ml of *C. albicans*).

### *In vitro* biofilm model

Biofilms were formed on optical microwell Petri dishes (MaTtek Corp, USA) that have a cover slip at the center to facilitate confocal microscopy. Single species biofilms were developed by incubating suspensions of *S. epidermidis* or *C. albicans* (O.D. 0.3) and mixed species biofilms by equal half volumes of both the organism suspensions, for 24 hr. Supernatants were discarded, biofilms washed with PBS, stained with LIVE/DEAD stain (Molecular Probes, USA). Bacteria with intact cell membranes (live cells) are stained green and those with damaged membranes, red. Biofilms were examined by the Nikon A1 confocal microscope (Nikon Instruments Inc., NY, USA) using fluorescein (green) and Texas red (red) band pass filter sets. Confocal images were obtained in serial sections at 1 μm intervals along the z-axis (40× magnification). The z-stack images were analyzed using software PHLIP in the MATLAB image processing toolbox, for biofilm biovolume (in μm^3^) [47].

### Mouse model of subcutaneous catheter biofilm infection

The protocol for animal experiments was approved by The Institutional Animal Care and Use Committee at Baylor College of Medicine. A biofilm infection model in mice with subcutaneously implanted catheters described previously was used [24]. Teflon catheters (Surflo, Terumo Corporation, Japan) sized 18G, 1½″ were pre-incubated in *S. epidermidis*, *C. albicans* or both organism suspensions (O.D. 0.3) for 2 hr, in order to facilitate biofilm development. Catheter segments were inserted subcutaneously in 3 week old weaned FVB albino mice. Catheter cultures were performed prior to subcutaneous insertion in serial dilution plating after 24 hr of incubation. Pre-insertion, catheters in suspensions of *S. epidermidis* yielded 3.5 to 4.5 × 10^5^ CFU/ml, those in *C. albicans* yielded 6 to 6.5 to 10^4^ CFU/ml and catheters immersed in mixed species suspensions yielded 1.5 to 2 × 10^4^ and 6 to 6.5 to 10^3^ of *S. epidermidis* and *C. albicans* respectively. Animals were euthanized on day 8; catheter and blood cultures were evaluated quantitatively for the two organisms and catheter biofilms examined by scanning electron microscopy.

#### Scanning electron microscopy

A 5 mm sample was cut from each explanted catheter segment from mice with subcutaneous catheter infection. The catheter samples were cut in cross sections and fixed with 2% glutaraldehyde, followed by fixation with osmium tetroxide, tannic acid and uranyl acetate. Fixation was followed by a series of ethanol dehydration steps and samples were sputter-coated with gold palladium. The samples were then scanned by electron microscopy for biofilms at different degrees of magnification.

### Microarrays

#### Cultures and RNA isolation for microarrays

Single species biofilms of *S. epidermidis* (strain 1457) and *C. albicans* (strain 32354) and mixed species biofilms were formed on 6-well tissue culture plates. Five ml of organism suspensions (O.D. 0.3, *S. epidermidis* 10^7^ CFU/ml or *C. albicans* 10^5^ CFU/ml) or 2.5 ml each for mixed-species biofilms for 24 hr. RNA was harvested from single species and mixed-species biofilms using RNeasy Mini kit (Qiagen) and Fast-RNA Pro-BLUE kit (MP Biomedicals) according to manufacturer’s instructions. Total RNA from 3 biological replicates each for *S. epidermidis* and mixed species biofilms was shipped to Mycroarray (http://www.mycroarray.com, Ann Arbor, USA) for hybridization to microarrays.

#### Microarray design

*In situ* synthesized oligonucleotide microarrays were manufactured by Mycroarray and probe sequence designed using a proprietary version of OligoArray 2.0 [48]. Arrays were synthesized on slide-sized glass substrates and each slide had an array composed of 40,962 spots, of which 33,715 spots contain 45mer probes for *S. epidermidis* genes, 525 empty features without a probe and 720 features with Mycroarray quality control probes. In addition, there are 6000 probes for randomly selected Candida genes to assess potential cross hybridization with *S. epidermidis* genes. There were up to 3 probes per gene and 5 identical replicates of each *S. epidermidis* probe. Multiple probes per gene format was chosen to account for the genetic variability between *S. epidermidis* 1457 strain used in our experiment compared to strain RP62A used in the microarray probe design. Also, to avoid theoretical cross contamination, *S. epidermidis* probes were blasted against *C. albicans* genome sequence (http://www.candidagenome.org) and *S. epidermidis* probes with potential match with *C. albicans* sequences were removed from the array design. Separately, RNA from pure *C. albicans* cultures were also hybridized to the arrays and cross-hybridizing probes were removed from data analysis.

#### Microarray hybridization and data analyses

Microarray experiments were performed by Mycroarray and data analyzed at Texas Children’s Hospital. Briefly, the purified mRNA was amplified and incorporated with amino allyl-UTP for indirect labeling with fluorescent dyes. Dynamic hybridization to the 40 K arrays was performed using Agilent gasket slides and incubation at 45°C for 20 hr following which the slides were washed and scanned using Axon 4000B scanner (Molecular Devices) at 5 micron resolution. Signal intensity values were extracted from scanned images using GenePix® Pro 6 software (Molecular Devices). The raw gpr files were loaded in Genespring GX 11.5, the data log_2_ transformed; background corrected, and normalized using the Quantile algorithm. Hierarchical clustering map was generating using Euclidean algorithm with the average linkage rule. Differential gene expression between the two samples groups (*S. epidermidis* and mixed species biofilms) was evaluated by unsupervised unpaired t-test on the log_2_ transformed mean data. A fold-change ratio (mixed species biofilms vs. *S. epidermidis* biofilms) was calculated with a fold change cutoff of 1.5 and p-value of 0.05. Probe set lists were trimmed to represent *S. epidermidis* and analyzed using unpaired t-test and Benjamini-Hochber multiple-testing correction to generate targeted lists of differential expression. Microarray expression patterns were validated using real-time PCR using three upregulated and two down regulated genes.

### Quantitation of eDNA in single and mixed-species biofilms

Biofilm matrix and eDNA were extracted from 24 hr single species *S. epidermidis* biofilms and mixed species biofilms of *S. epidermidis* and *C. albicans* as described previously [30,39,46]. The extracellular matrix from harvested biofilms was carefully extracted without cell lysis and contamination with genomic DNA as described [30,39,46]. The amount of eDNA was quantified by real-time RT-PCR using standard curves of known quantities of *S. epidermidis* and *C. albicans* genomic DNA. Real-time PCR was performed using the SYBR Green kit (Qiagen) and primers for 3 chromosomal genes of *S. epidermidis*, *lrgA*, *lrgB* and *bap* (whose primers for RT-PCR were previously optimized in our lab) or stably expressed chromosomal genes of *C. albicans RIP, RPP2B and PMA1*[49]. The amount of measured eDNA was normalized for 10^8^ CFU organisms in the initial inoculation.

### Effects of DNAse on single and mixed species biofilms

Concentration dependent effects of DNAse I (Sigma or Roche, USA) was studied by exposing 24 hr single and mixed-species biofilms, at 0 to 1.25 mg/ml concentrations DNAse I for 16 hr and residual biofilm evaluated by measuring absorbance at 490 nm after XTT reduction [50]. A time course experiment was performed by the addition of DNAse (0.65 mg/ml) at 0, 6 or 18 hrs of biofilm development. The biofilms were developed for a total of 24 hr and metabolic activity quantitated by XTT method and measuring absorbance at 490 nm. Percentage reduction in biofilms compared to controls was evaluated for single and mixed species biofilms at DNAse exposures starting at 0, 6 or 18 hrs.

### Data deposition

The microarray dataset supporting the results of this article has been deposited and available at the NCBI gene expression and hybridization data repository (http://www.ncbi.nlm.nih.gov/geo/), [GEO accession number GSE35438].

## Abbreviations

CONS: Coagulase-negative staphylococci; eDNA: Extracellular DNA; ECM: Extracellular matrix; O.D.: Optical density; CFU/ml: Colony forming units/ml; RT-PCR: Reverse transcriptase polymerase chain reaction; TSB: Tryptic soy broth; SDA: Sabouraud’s dextrose agar; YPD: Yeast peptone dextrose.

## Competing interests

None of authors have competing financial or non-financial interests associated with this article.

## Authors’ contributions

MP conceived the project, did the biofilm experiments *in vitro* and *in vivo* including imaging studies, analyzed data and wrote the manuscript. RL assisted in the biofilm experiments, *in vitro* and *in vivo* experiments and imaging studies. JH performed the electron microscopy studies of the catheter biofilms. TM carried out the microarray analyses and participated in the revision of the manuscript. JM contributed by critical intellectual input and revision of the manuscript. All authors read and approved the final manuscript.

## Authors’ information

Mohan Pammi MD

Assistant Professor and Neonatologist, Baylor College of Medicine and Texas Children’s Hospital, Houston, Texas.

Rong Liang MD

Research Associate, Baylor College of Medicine and Texas Children’s Hospital, Houston, Texas.

John Hicks MD PhD

Professor of Pathology, Baylor College of Medicine and Texas Children’s Hospital, Houston, Texas.

Toni-Ann Mistretta PhD

Senior Biostatistician, Baylor College of Medicine & Texas Children's Microbiome Center

James Versalovic MD PhD

Professor and Chief of the Department of Pathology, Baylor College of Medicine and Texas Children’s Hospital, Houston, Texas.

## Supplementary Material

Additional file 1: Table S1 Differential expression of *S. epidermidis* genes in mixed-species biofilms. Click here for file
