# Supplementary material for: Biofilm extracellular DNA enhances mixed species biofilms of Staphylococcus epidermidis and Candida albicans
Source: BMC Microbiol. 2013 Nov 14;13:257. doi: 10.1186/1471-2180-13-257 (PMC3833181; doi:10.1186/1471-2180-13-257)
Supplement: Additional file 1: Table S1 — Differential expression of S. epidermidis genes in mixed-species biofilms. [file 1471-2180-13-257-S1.doc]

**Supplemental Table 1. Differential expression of *S. epidermidis* genes in mixed-species biofilms**

| **1A. *S. epidermidis* genes upregulated in mixed-species biofilms with *C. albicans*** | | | |
| --- | --- | --- | --- |
|  | **Gene product name** | **Gene ID** | **Fold change** |
|  | **I. TRANSCRIPTIONAL REGULATORS** |  |  |
| *sarR* | Accessory regulator R | SERP 1876 | 2.1 |
| *HrcA* | Heat-inducible transcription repressor HrcA | SERP 1150 | 1.9 |
|  | PemK family transcriptional regulator | SERP 1681 | 1.7 |
|  | Accessory regulator V | SERP 1849 | 1.7 |
| *rho* | Transcription termination factor Rho | SERP 1728 | 1.7 |
| *HrcA* | Heat-inducible transcription repressor HrcA | SERP 1150 | 1.6 |
| *VraR* | DNA-binding response regulator VraR | SERP 1422 | 1.5 |
|  |  |  |  |
|  | **II. HEAT SHOCK PROTEINS** |  |  |
| *grpE* | Heat shock protein GrpE | SERP 1149 | 2.1 |
|  |  |  |  |
|  | **III. TRANSPORTERS AND RECEPTORS** |  |  |
|  | ABC transporter, substrate-binding protein | SERP 2383 | 2.2 |
|  | Transferrin receptor | SERP 0949 | 2.1 |
| *gltS* | Sodium:glutamate symporter | SERP 1935 | 1.8 |
| *potD* | Spermidine/putrescine ABC transporter, spermidine/putrescine-binding protein | SERP 0689 | 1.6 |
| *potC* | Spermidine/putrescine ABC transporter, permease protein PotC | SERP 0688 | 1.5 |
|  | Peptide ABC transporter, ATP-binding protein | SERP 2369 | 1.5 |
|  |  |  |  |
|  | **IV. METABOLISM** |  |  |
| *guaC* | Guanosine monophosphate reductase | SERP 0906 | 3.2 |
| *purC* | 5-phospho-D-ribosyl imidazole-4-carboxylate and L-aspartate in purine biosynthesis; SAICAR synthase | SERP 0651 | 2.9 |
| *glpD* | Aerobic glycerol-3-phosphate dehydrogenase | SERP 0868 | 2.6 |
| *apt* | Adenine phosphoribosyltransferase | SERP 1198 | 2.8 |
| *uraA* | Uracil permease | SERP 0765 | 2.4 |
| *purM* | Phosphoribosylaminoimidazole synthetase | SERP 0656 | 2.6 |
|  | IS431mec-like transposase | SERP 1579 | 2.2 |
|  | 7-cyano-7-deazaguanine reductase | SERP 0394 | 1.9 |
| *prmA* | Ribosomal protein L11 methyltransferase | SERP 1146 | 1.7 |
| *deoD* | Purine nucleoside phosphorylase | SERP 1747 | 1.6 |
|  | NADH dehydrogenase, putative | SERP 0524 | 1.6 |
|  |  |  |  |
|  | **V. OTHER PROTEINS** |  |  |
| *prfA* | Peptide chain release factor 1 | SERP 1725 | 3.6 |
| *HrcA* | Heat-inducible transcription repressor HrcA | SERP 1150 | 3.5 |
| *femX* | FemX protein | SERP 1844 | 3.0 |
|  | Holliday junction resolvase-like protein | SERP 1180 | 2.7 |
| *fmt* | Fmt protein | SERP 0641 | 2.4 |
| *rpsN* | 30S ribosomal protein S14 | SERP 0905 | 2.2 |
|  | Putative lipoprotein | SERP 2167 | 2.1 |
|  | Radical SAM domain-containing protein | SERP 1415 | 2.1 |
|  | PAP2 family protein | SERP 0992 | 1.8 |
| *mraZ* | Cell division protein MraZ | SERP 0743 | 1.8 |
|  | AIR carboxylase, putative | SERP 0254 | 1.8 |
|  | Competence protein ComGA, putative | SERP 1109 | 1.6 |
|  | Rhodanese-like domain-containing protein | SERP 1317 | 1.6 |
|  |  |  |  |
|  | **IV. HYPOTHETICAL PROTEINS** |  |  |
|  | Hypothetical protein | SERP 2171 | 11.0 |
|  | Conserved hypothetical protein | SERP 0224 | 7.4 |
|  | Hypothetical protein | SERP 2013 | 3.5 |
|  | Hypothetical protein | SERP 1216 | 3.3 |
|  | Hypothetical protein | SERP 0438 | 3.3 |
|  | Hypothetical protein | SERP 1216 | 3.2 |
|  | Conserved hypothetical protein | SERP 1469 | 3.1 |
|  | Conserved hypothetical protein | SERP 0159 | 3.1 |
|  | Conserved hypothetical protein | SERP 0272 | 3.0 |
|  | Hypothetical protein | SERP 0880 | 3.0 |
|  | Conserved hypothetical protein | SERP 0272 | 2.8 |
|  | Hypothetical protein | SERP 1179 | 2.7 |
|  | Hypothetical protein | SERP 0907 | 2.5 |
|  | Hypothetical protein | SERP 1303 | 2.4 |
|  | Hypothetical protein | SERP 0907 | 2.4 |
|  | Hypothetical protein | SERP 2256 | 2.4 |
|  | Hypothetical protein | SERP 0910 | 2.3 |
|  | Hypothetical protein | SERP 1029 | 2.3 |
|  | Hypothetical protein | SERP 0859 | 2.2 |
|  | Hypothetical protein | SERP 0907 | 2.2 |
|  | Conserved hypothetical protein | SERP 1425 | 2.1 |
|  | Hypothetical protein | SERP 2271 | 2.0 |
|  | Hypothetical protein | SERP 0910 | 1.7 |
|  | Hypothetical protein | SERP 2223 | 1.7 |
|  | Conserved hypothetical protein | SERP 0787 | 1.7 |
|  | Hypothetical protein | SERP 0294 | 1.7 |
|  | Hypothetical protein | SERP 0294 | 1.6 |
|  | Hypothetical protein | SERP 2148 | 1.6 |
|  | Conserved hypothetical protein | SERP 0121 | 1.9 |

| **1B. *S. epidermidis* genes down- regulated in mixed-species biofilms with *C. albicans*** | | | |
| --- | --- | --- | --- |
|  | 1. **AUTOLYSIS AND CELL DEATH** |  |  |
| *lrgA* | Holin-like protein LrgA | SERP 2026 | -36.1 |
| *lrgB* | LrgB protein | SERP 2027 | -27.0 |
|  |  |  |  |
|  | 1. **METABOLISM** |  |  |
|  | **Carbohydrate metabolism** |  |  |
| *garK* | Glycerate kinase 2 | SERP 2012 | -2.8 |
| *gntP* | Gluconate transporter, permease protein | SERP 2057 | -2.8 |
| *gntK* | Gluconokinase | SERP 2058 | -2.7 |
| *gntR* | Gluconate operon transcriptional repressor | SERP 2059 | -2.7 |
|  | Acetyl-CoA acetyltransferase, putative | SERP 0220 | -2.6 |
| *nuoF* | NADH dehydrogenase I, F subunit | SERP 0084 | -2.5 |
|  | Acetyltransferase, GNAT family | SERP 2059 | -2.4 |
| *nagA* | N-acetylglucosamine-6-phosphate deacetylase | SERP 0360 | -2.4 |
| *gntK* | Gluconokinase | SERP 2058 | -2.4 |
|  | Bifunctional acetaldehyde-CoA/alcohol dehydrogenase | SERP 0389 | -2.3 |
| *sdhC* | Succinate dehydrogenase, cytochrome b558 | SERP 0730 | -2.2 |
| *gldA* | Glycerol dehydrogenase | SERP 2346 | -2.2 |
|  | Acetyltransferase | SERP 2082 | -1.8 |
|  | Alcohol dehydrogenase, zinc-containing | SERP 1785 | -1.8 |
|  | Acetyl-CoA carboxylase, biotin carboxyl carrier | SERP 1170 | -1.7 |
|  | Alcohol dehydrogenase, zinc-containing | SERP 1785 | -1.7 |
|  | Acetyltransferase | SERP 1963 | -1.7 |
|  | Acetyltransferase | SERP 1863 | -1.6 |
| *thiE* | Thiamine-phosphate pyrophosphorylase | SERP 1698 | -1.6 |
| *est* | Carboxylesterase | SERP 0449 | -1.6 |
|  |  |  |  |
|  | **Aminoacid metabolism** |  |  |
| *thrB* | Homoserine kinase | SERP 0899 | -2.9 |
|  | Aminotransferase, class II | SERP 2394 | -2.8 |
| *mtn* | 5'-methylthioadenosine/S-adenosylhomocysteine nucleosidase | SERP 1166 | -2.7 |
| *thrC* | Threonine synthase | SERP 0898 | -2.5 |
| *ilvE* | Branched-chain amino acid aminotransferase | SERP 0195 | -2.3 |
|  | Tyrosine recombinase XerC | SERP 0818 | -2.3 |
| *gltD* | Glutamate synthase, small subunit | SERP 0109 | -2.1 |
| *msrA2* | Methionine sulfoxide reductase A | SERP 1000 | -2 |
| *leuA* | 2-isopropylmalate synthase | SERP 1669 | -2 |
| *leuC* | Isopropylmalate isomerase large subunit | SERP 1671 | -2 |
| *est* | Carboxylesterase | SERP 0449 | -2 |
| *XerC* | Tyrosine recombinase XerC | SERP 0818 | -1.9 |
| *msrA2* | Methionine sulfoxide reductase A | SERP 1000 | -1.8 |
| *leuS* | Leucyl-tRNA synthetase | SERP 1318 | -1.7 |
|  | Carboxylesterase, putative | SERP 0090 | -1.6 |
| *panD* | Aspartate alpha-decarboxylase | SERP 2150 | -1.5 |
| *ilvC* | Ketol-acid reductoisomerase | SERP 1668 | -1.5 |
|  | Cytosol aminopeptidase | SERP 0528 | -1.5 |
|  |  |  |  |
|  | **Nucleic acid metabolism** |  |  |
| *mfd* | Transcription-repair coupling factor | SERP 0141 | -3.5 |
| *nth* | Endonuclease III | SERP 1022 | -2.4 |
|  | RNA polymerase sigma factor sigW, putative | SERP 0174 | -2.2 |
|  | Ribonuclease BN, putative | SERP 1421 | -2.1 |
| *cysS* | Cysteinyl-tRNA synthetase | SERP 0170 | -2 |
| *ruvB* | Holliday junction DNA helicase RuvB | SERP 1205 | -1.9 |
|  | DNA polymerase III, epsilon subunit/ATP-dependent helicase DinG | SERP 1025 | -1.6 |
| *dnaB* | Replicative DNA helicase | SERP 2537 | -1.6 |
|  | RNA methyltransferase | SERP 0172 | -1.6 |
|  |  |  |  |
|  | **Other metabolism** |  |  |
| *cls-2* | Cardiolipin synthetase | SERP 1695 | -3.5 |
|  | Oxidoreductase, short chain | SERP 1917 | -3.4 |
|  | Oxidoreductase, short-chain | SERP 2129 | -2.3 |
| *hemD* | Uroporphyrinogen-III synthase | SERP 1233 | -2.1 |
|  | 2-dehydropantoate 2-reductase PanE, putative | SERP 2022 | -2.1 |
|  | O-succinylbenzoic acid synthetase, putative | SERP 1357 | -2 |
| *leuB* | 3-isopropylmalate dehydrogenase | SERP 1670 | -2 |
| *coaD* | Phosphopantetheine adenyltransferase | SERP 0715 | -1.8 |
|  | 2-oxoisovalerate dehydrogenase, E1 component, beta subunit" | SERP 1077 | -1.8 |
| *hemC* | Porphobilinogen deaminase | SERP 1234 | -1.7 |
| *coaBC* | Phosphopantothenoylcysteine decarboxylase/phosphopantothenate--cysteine ligase | SERP 0778 | -1.7 |
|  |  |  |  |
|  | **III. TRANSPORTERS AND RECEPTORS** |  |  |
| *sitA* | ABC transporter, ATP-binding protein | SERP 0292 | -2.8 |
| *oppF* | Oligopeptide ABC transporter, ATP-binding | SERP 0573 | -2.4 |
|  | Amino acid permease family protein | SERP 2142 | -2.3 |
|  | Aminotransferase, class V | SERP 1287 | -2.3 |
| *mnhA* | Monovalent cation/H+ antiporter subunit A | SERP 0538 | -2.2 |
| *oppC* | Oligopeptide ABC transporter, permease protein | SERP 0571 | -2.1 |
| *oppB* | Oligopeptide ABC transporter, permease protein | SERP 0570 | -2.1 |
| *oppD* | Oligopeptide ABC transporter, ATP-binding | SERP 0572 | -2 |
| *pbuX* | Xanthine permease | SERP 0068 | -1.8 |
|  | ABC transporter, permease protein | SERP 1005 | -1.7 |
|  | ABC transporter, ATP-binding protein | SERP 2204 | -1.6 |
|  | Transporter, putative | SERP 2023 | -1.5 |
| *mscL* | Large-conductance mechanosensitive channel | SERP 0929 | -1.5 |
|  |  |  |  |
|  | **IV. OTHER PROTEINS** |  |  |
|  | CBS domain protein | SERP 0361 | -5.7 |
|  | Abortive infection family protein | SERP 1486 | -3.3 |
|  | Cation efflux family protein | SERP 0465 | -3.2 |
|  | Inosine-uridine preferring nucleoside hydrolase family protein | SERP 1865 | -2.9 |
|  | ATP-binding protein, Mrp/Nbp35 family | SERP 1765 | -2.8 |
|  | Surface lipoprotein-related protein | SERP 0882 | -2.7 |
| *yycJ* | Metallo-beta-lactamase family protein YycJ | SERP 2530 | -2.7 |
|  | LysM domain protein | SERP 0318 | -2.6 |
|  | Cell cycle protein FtsW | SERP 1691 | -2.6 |
| *ypfP* | ypfP protein | SERP 0606 | -2.5 |
|  | Glyoxalase family protein | SERP 0760 | -2.4 |
| *rplL* | Ribosomal protein L7/L12 | SERP 0181 | -2.4 |
|  | Cation efflux family protein | SERP 0465 | -2.3 |
|  | Cell cycle protein FtsW | SERP 1691 | -2.3 |
|  | CBS domain protein | SERP 0507 | -2.3 |
|  | ComE operon protein 2 | SERP 1156 | -2.2 |
|  | LysM domain protein | SERP 0318 | -2.1 |
|  | LysM domain protein | SERP 0100 | -2.1 |
|  | Chorismate | SERP 1297 | -2 |
| *rplJ* | 50S ribosomal protein L10 | SERP 0180 | -2 |
|  | Alkaline shock protein 23 | SERP 1782 | -1.9 |
|  | Glyoxalase family protein | SERP 0760 | -1.9 |
|  | Phosphoglucomutase/phosphomannomutase family protein | SERP 2055 | -1.8 |
| *xpaC* | xpaC protein, putative | SERP 0975 | -1.8 |
|  | CrcB family protein | SERP 1339 | -1.8 |
|  | Pyridine nucleotide-disulfide oxidoreductase family protein | SERP 0242 | -1.8 |
| *hemX* | hemX protein | SERP 1235 | -1.8 |
| *xerC* | DNA processing protein DprA, putative | SERP 0815 | -1.7 |
|  | Decarboxylase family protein | SERP 0335 | -1.7 |
|  | CBS domain protein | SERP 0507 | -1.7 |
|  | Cell wall surface anchor family protein | SERP 0719 | -1.6 |
|  | Myosin-cross-reactive antigen | SERP 0663 | -1.6 |
| *fabF* | 3-oxoacyl-(acyl-carrier-protein) synthase II | SERP 0568 | -1.6 |
| *DnaI* | Primosomal protein DnaI | SERP 1247 | -1.6 |
| *PhoU* | Phosphate transport system regulatory protein PhoU, putative | SERP 0956 | -1.6 |
|  | Tellurite resistance protein, putative | SERP 0976 | -1.6 |
| *frr* | Ribosome recycling factor | SERP 0826 | -1.5 |
|  | Sensor histidine kinase | SERP 1954 | -1.5 |
|  |  |  |  |
|  | **V. Hypothetical proteins** |  |  |
|  | Hypothetical protein | SERP 0693 | -3.9 |
|  | Conserved hypothetical protein | SERP 0079 | -3.7 |
|  | Conserved hypothetical protein | SERP 1053 | -3.5 |
|  | Conserved hypothetical protein | SERP 0336 | -3.2 |
|  | Hypothetical protein | SERP 1167 | -3 |
|  | Conserved hypothetical protein TIGR00159 | SERP 1764 | -2.7 |
|  | Hypothetical protein | SERP 1053 | -2.6 |
|  | Hypothetical protein | SERP 2044 | -2.5 |
|  | Hypothetical protein | SERP 1998 | -2.5 |
|  | Conserved hypothetical protein | SERP 0261 | -2.4 |
|  | Hypothetical protein | SERP 0301 | -2.4 |
|  | Conserved hypothetical protein | SERP 0336 | -2.4 |
|  | Conserved hypothetical protein | SERP 1140 | -2.1 |
|  | Conserved hypothetical protein | SERP 0319 | -2 |
|  | Conserved hypothetical protein | SERP 0106 | -2 |
|  | Hypothetical protein | SERP 1365 | -1.9 |
|  | Hypothetical protein | SERP 1031 | -1.8 |
|  | Hypothetical protein | SERP 0997 | -1.8 |
|  | Hypothetical protein | SERP 0388 | -1.8 |
|  | Hypothetical protein | SERP 0634 | -1.7 |
|  | Hypothetical protein | SERP 1365 | -1.7 |
|  | Hypothetical protein | SERP 1687 | -1.7 |
|  | Hypothetical protein | SERP 1207 | -1.7 |
|  | Hypothetical protein | SERP 1207 | -1.7 |
|  | Hypothetical protein | SERP 1052 | -1.7 |
|  | Hypothetical protein | SERP 1687 | -1.7 |
|  | Hypothetical protein | SERP 1031 | -1.6 |
|  | Hypothetical protein | SERP 1778 | -1.6 |
|  | Hypothetical protein | SERP 0639 | -1.6 |
